# Compound heterozygous *IFT140* variants in two Polish families with Sensenbrenner syndrome and early onset end-stage renal disease

**DOI:** 10.1186/s13023-020-1303-2

**Published:** 2020-02-01

**Authors:** Joanna Walczak-Sztulpa, Renata Posmyk, Ewelina M. Bukowska-Olech, Anna Wawrocka, Aleksander Jamsheer, Machteld M. Oud, Miriam Schmidts, Heleen H. Arts, Anna Latos-Bielenska, Anna Wasilewska

**Affiliations:** 1 Department of Medical Genetics, Poznan University of Medical Sciences, Rokietnicka 8 Street, 60-608 Poznan, Poland; 2 Department of Perinatology, Medical University of Bialystok, Bialystok, Poland; 3 Department of Human Genetics, Radboud University Medical Center, Nijmegen, The Netherlands; 4 Radboud Institute for Molecular Life Sciences, Radboud University Medical Centre, Nijmegen, The Netherlands; 5 Center for Pediatrics and Adolescent Medicine, Freiburg University Hospital, Freiburg University Faculty of Medicine, Freiburg, Germany; 6 Department of Pathology and Laboratory Medicine, Dalhousie University, Halifax, Nova Scotia Canada; 7 IWK Health Centre, Clinical Genomics Laboratory, Halifax, Nova Scotia Canada; 8 Department of Pediatrics and Nephrology, Medical University of Bialystok, Bialystok, Poland

**Keywords:** Sensenbrenner syndrome, Cranioectodermal dysplasia, *IFT140*, Ciliopathy, End-stage renal disease

## Abstract

**Background:**

Sensenbrenner syndrome, which is also known as cranioectodermal dysplasia (CED), is a rare, autosomal recessive ciliary chondrodysplasia characterized by a variety of clinical features including a distinctive craniofacial appearance as well as skeletal, ectodermal, liver and renal anomalies. Progressive renal disease can be life-threatening in this condition. CED is a genetically heterogeneous disorder. Currently, variants in any of six genes (*IFT122, WDR35, IFT140, IFT43, IFT52* and *WDR19*) have been associated with this syndrome. All of these genes encode proteins essential for intraflagellar transport (IFT) a process that is required for cilium assembly, maintenance and function. Intra- and interfamilial clinical variability has been reported in CED, which is consistent with CED’s genetic heterogeneity and is indicative of genetic background effects.

**Results:**

Two male CED patients from two unrelated Polish families were included in this study. Clinical assessment revealed distinctive clinical features of Sensenbrenner syndrome, such as dolichocephaly, shortening of long bones and early onset renal failure. Ectodermal anomalies also included thin hair, short and thin nails, and small teeth in both patients. Next generation sequencing (NGS) techniques were performed in order to determine the underlying genetic cause of the disorder using whole exome sequencing (WES) for patient 1 and a custom NGS-based panel for patient 2. Subsequent qPCR and duplex PCR analysis were conducted for both patients. Genetic analyses identified compound heterozygous variants in the *IFT140* gene in both affected individuals. Both patients harbored a tandem duplication variant p.Tyr1152_Thr1394dup on one allele. In addition, a novel missense variant, p.(Leu109Pro), and a previously described p.(Gly522Glu) variant were identified in the second allele in patients 1 and 2, respectively. Segregation analysis of the variants was consistent with the expected autosomal recessive disease inheritance pattern. Both patients had severe renal failure requiring kidney transplantation in early childhood.

**Conclusion:**

The finding of compound heterozygous *IFT140* mutations in two unrelated CED patients provide further evidence that *IFT140* gene mutations are associated with this syndrome. Our studies confirm that *IFT140* changes in patients with CED are associated with early onset end-stage renal disease. Moreover, this report expands our knowledge of the clinical- and molecular genetics of Sensenbrenner syndrome and it highlights the importance of multidisciplinary approaches in the care of CED patients.

## Background

Sensenbrenner syndrome is an ultra-rare autosomal recessive disorder that is thought to result from dysfunction of cilia. CED is a genetically heterogeneous disease affecting multiple systems. It is diagnosed based on characteristic clinical features, which include sagittal craniosynostosis, dolichocephaly, facial dysmorphisms such as epicanthal folds, telecanthus, hypertelorism and frontal bossing, growth retardation, shortening of upper and lower limbs, narrow thorax, protuberant abdomen, progressive renal disease and ectodermal abnormalities. Liver and retinal dysfunction have also been reported in CED patients, albeit less frequently [1, 2].

To date, more than 60 patients have been reported in literature and mutations in six genes have been associated with Sensenbrenner syndrome: *IFT122, WDR35, IFT140, IFT43, IFT52* and *WDR19* [3–8]. All of these genes encode proteins that are involved in intraflagellar transport (IFT). This is a bi-directional transport process that occurs in the cilium and plays a crucial role in cilium assembly, maintenance and function. The IFT140 protein is part of the so-called IFT-A complex that primarily regulates retrograde intraflagellar ciliary transport (i.e., transport of cargo proteins from the ciliary tip to its base)*.* The *IFT140* gene consists of 31 exons (29 coding exons) and encodes a 1462 amino acid protein that contains five WD repeats and nine tetratricopeptide (TPR) repeats [9–11].

Mutations in *IFT122* and *WDR35* are the most common cause of CED and explain about 60% of families with Sensenbrenner syndrome. To date, only two unrelated CED patients with *IFT140* variants have been reported in literature [8, 12]. *IFT140* mutations thus constitute a relatively uncommon cause of CED. However, clinical phenotypes associated with dysfunction of IFT140 are not limited to CED. In fact, pathogenic variation in *IFT140* has also been reported in patients with Mainzer-Saldino Syndrome (MSS), Jeune Syndrome (JATD), Opitz trigonocephaly C syndrome (OTCS), and isolated retinal dystrophy [13, 14]. CED, MSS and JATD are phenotypically and genetically related disorders, and are collectively referred to as short-rib thoracic dysplasia syndromes [15]. The identification of variants in *IFT140* in patients with various, overlapping phenotypic features is in line with the general presumption that ciliopathies represent a spectrum of disorders with marked phenotypic and genotypic overlap among distinctly-classified conditions.

### Clinical examination

In our study two male patients from two unrelated, non-consanguineous Polish families were diagnosed with Sensenbrenner syndrome.

#### Patient 1

A 3.5-years-old boy was referred to the Genetic Counseling Unit due to renal failure, skeletal abnormalities and ocular problems. The patient was born to young (20-year-old mother and 33-year-old father), unrelated parents from a first, unremarkable pregnancy, by conventional vaginal delivery in the 39th week of gestation. The birth weight was 3700 g (50th -75th centile), length 57 cm (97th centile), head circumference 36 cm (75th–97th centile), and thorax circumference 33 cm (25-50th centile). The Apgar score was 9 at 1 min. The family history was unremarkable. Although the family doctor recorded proteinuria, hematuria and glycosuria in the early neonatal period, no further evaluation was conducted. At the age of 12 months the child was treated for a urinary tract infection. At three years of age, he developed bronchopneumonia and was hospitalized in a district hospital, where elevated creatinine levels were detected. The child was referred to the Pediatric Nephrology Department for further diagnosis. Routine laboratory investigations showed constant proteinuria, glycosuria, hematuria, eGFR (Schwartz)- 30,56 ml/min/1.73 m2, elevated serum levels of creatinine, uric acid and urea, total cholesterol, and triglyceride were determined, whereas complete blood count, blood glucose, albumin, thyroid and liver function tests were normal. Ultrasonography of kidneys showed increased renal cortex echogenicity and decreased cortico-medullary differentiation.

**Fig. 1:**
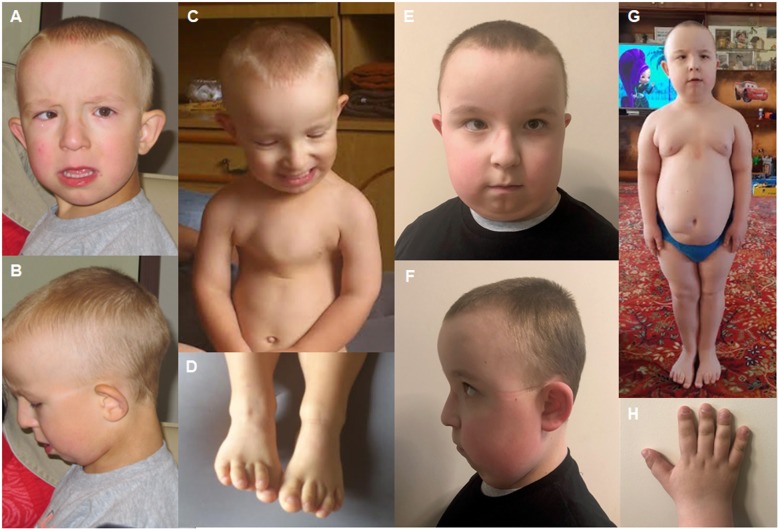
Dysmorphic features and changing phenotype of patient 1 at the age of 3 y 7 m (**a**-**d**) and 9 y 3 m (**e**-**h**) respectively. (**a**, **e**) Characteristic facial features. Facial features are dolichocephaly, high prominent forehead, thin sparse hair, full cheeks, strabismus, broad prominent nasal bridge, flat *philtrum* columns, narrow mucus upper lip, everted lower lip, open mouth, low-set prominent ears, microretrognathia. (**b**, **f**) A facial lateral view showing low set ears and sparse hair. (**c**) Narrow chest, pectus excavatum. (**d**) Brachydactyly and overlap of toes. (**g**) Whole body image showing proximal limb shortening, a short broad neck, a narrow thorax and obesity. (**h**) Brachydactyly of the right hand

Distinctive dysmorphic features were observed during the first assessment by a clinical geneticist at the age of 3.5 years. The features included dolichocephaly, high forehead, thin hair, full cheeks, low set prominent ears, long *philtrum,* microretrognathia, rhizomelic shortening of upper and lower limbs, brachydactyly of toes and fingers, narrow chest and pectus excavatum. These features, in combination with progressive renal failure, were indicative of a ciliopathy (Fig. 1 a-d and Table 1). Ophthalmological examination confirmed strabismus, nystagmus and high hyperopia. Cytogenetic analysis (conventional GTG banding) revealed a normal male karyotype (46,XY). The patient was seen in the genetics clinic every 6 months. Renal function rapidly declined in the following 12 months. At the age of 4.5 years the patient had developed end-stage renal failure and peritoneal dialysis was ordered. At the age of 6.5 years, his weight was 19.3 kg (25th–50th centile), his height was 102 cm (< 3rd centile), and his BMI was 18,55 kg/m2 (97th centile). His morphological phenotype markedly differed from the previous clinical assessments during the last examination at the age of 9 years (Fig. 1 e-h). Short stature, obesity, short fingers and toes, and shortening of the long bones were more prominent. Dolichocephaly was less evident. Psychomotor development was normal. To date, he attends a normal primary school; he is a very intelligent and positively oriented boy.

**Table 1:** Clinical features of CED patients carrying compound heterozugous variants in *IFT140*

Patient	1	2
Variant (DNA)	c.326 T > C + c.3454-488_4182 + 2588dup	c.1565G > A + + c.3454-488_4182 + 2588dup
Variant (protein)	p.(Leu109Pro) + p.Tyr1152_Thr1394dup	p.(Gly522Glu) + p.Tyr1152_Thr1394dup
Age at examination	3 years 6 months	14 months
Sex	male	male
Dolichocephaly	+	+
Craniosynostosis	+	–
Frontal bossing	+	+
Full cheeks	+	+
Broad nasal bridge	+	+
Long philtrum	+	+
Flat philtrum columns	+	–
Narrow mucus upper lip	+	+
Everted lower lip	+	–
Low set ears	+	+
Protruding ears	+	+
Narrow chest	+	+
Pectus excavatum	+	–
Short ribs	+	+
Wide ribs	+	+
Rhizomelic limb shortening	+	+
Short humeri	+	+
Short fibulae	+	+
Flattened epiphyses	+	+
Brachydactyly of fingers and toes	+	+
Overlapping toes	+	–
Joint laxity	+	+
Fine, sparse hair	+	+
Small teeth	+	+
Thin nails	+	+
Short nails	+	+
Protuberant abdomen	+	+
Progressive renal failure	+	+
Tubulointerstitial nephritis	+	+
Ophthalmological problems	Hyperopia, strabismus, nystagmus	Hyperopia
Recurrent respiratory infections	+	+
Intelligence	Normal	Normal
Motor development	Normal	Delayed (walking at 22 months)

#### Patient 2

A 14-month-old male patient was referred to our clinic with suspicion of chondrodysplasia. The patient was born at 40 weeks of gestation, after a first, uneventful pregnancy. He was delivered by Cesarean section. Anthropometric measurements were in the normal range: his birth weight was 3500 g (50th–75th centile), his body length was 58 cm (97th centile), his head circumference was 34 cm (25th–50th centile), his thorax circumference was 33 cm (25th–50th centile) and he had an Apgar score of 10. His parents, a 28-year-old mother and a 33-year-old father, are healthy and have a non-consanguineous relationship. The family history was unremarkable. The neonatal period was complicated by recurrent respiratory infections, mild hypotonia, and atopic skin. The first renal problems, proteinuria and hematuria, were observed in the 7th month. The boy was hospitalized at 8th months and a full diagnostic assessment was performed. The ultrasound revealed bilateral enlarged kidneys (left 61 mm and right 63 mm) with edema and poor cortico-medullary differentiation. Renal biopsy showed a chronic injury of the parenchyma. Due to progressive renal failure and an advanced stage of renal insufficiency, peritoneal dialysis was initiated. Renal transplantation has been suggested for this patient. Anomalies of other internal organs were not identified. Hyperopia and nystagmus were noted during ophthalmological examination.

**Fig. 2:**
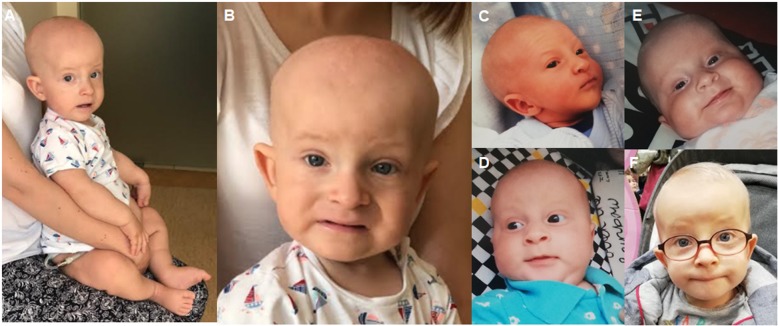
Dysmorphic features and changing phenotype of patient 2. At the age of 14 months (**a**, **b**). Facial features including dolichocephaly, high prominent forehead, “senile-like” face, very thin sparse hair, full cheeks, thin upper and lower lip, low-set protruding ears, pointed chin, small teeth. (**c**) Newborn period, (**d**) 2-month-old, (**e**) 4-month-old, (**f**) 23-month-old

The patient was first seen by a clinical geneticist at the age of 14 months. The geneticist noted that the patient had a short stature with rhizomelic shortening in upper and lower extremities. Distinctive dysmorphic features indicative of Sensenbrenner syndrome were found. These included dolichocephaly, high prominent forehead, “senile-like” face, very thin sparse hair, full cheeks, thin upper and lower lip, low-set protruding ears, pointed chin and small teeth (Fig. 2a-f and Table 1). Developmental milestones were delayed: the patient was able to sit independently at 12 months, but did not crawl. He started walking at 22 months and began to say a few simple words.

Patient 1 and patient 2 presented with very similar phenotypes and a clinical diagnosis of Sensenbrenner syndrome was made for both patients (Table 1).

## Results

### Genetic analysis

Next generation sequencing was performed for both patients in order to determine the molecular cause of CED. A whole-exome was sequenced in patient 1 and a custom panel of 61 genes and 11 SNPs was sequenced for patient 2. Genetic analyses were complemented by qPCR and duplex PCR analysis for both patients.

#### Patient 1

Whole exome sequencing (WES) revealed a heterozygous missense variant c.326 T > C; p.(Leu109Pro) in *IFT140*. Subsequent qPCR and duplex PCR analysis in a combination with Sanger sequencing showed a heterozygous tandem duplication c.3454-488_4182 + 2588dup; p.Tyr1152_Thr1394dup on the other allele. The variants were inherited from the patient’s unaffected father and mother, respectively. The tandem duplication was not identified through WES analysis.

The missense substitution p.(Leu109Pro) is a novel variant, which has not previously been reported in the HGMD database. The variant was not reported in the 1000 Genomes, the NHLBI Exome Variant Server (EVS), the Exome Aggregation Consortium (ExAC) or the gnomAD database (25.07.2019). This change was predicted to be pathogenic by MutationTaster, PolyPhen and SIFT software. We classified the p.(Leu109Pro) variant as likely pathogenic based on variant classification guidelines from the American College of Medical Genetics and Genomics and the Association for Molecular Pathology [16].

#### Patient 2

Sequencing of a custom gene panel by NGS revealed a heterozygous missense variant c.1565G > A; p.(Gly522Glu) in *IFT140* in the patient. Subsequent qPCR and duplex PCR analysis in combination with Sanger sequencing identified the same heterozygous tandem duplication that was observed in patient 1. The variants were inherited from the patient’s unaffected mother and father, respectively. This tandem duplication was not detected by NGS analysis.

**Fig. 3:**
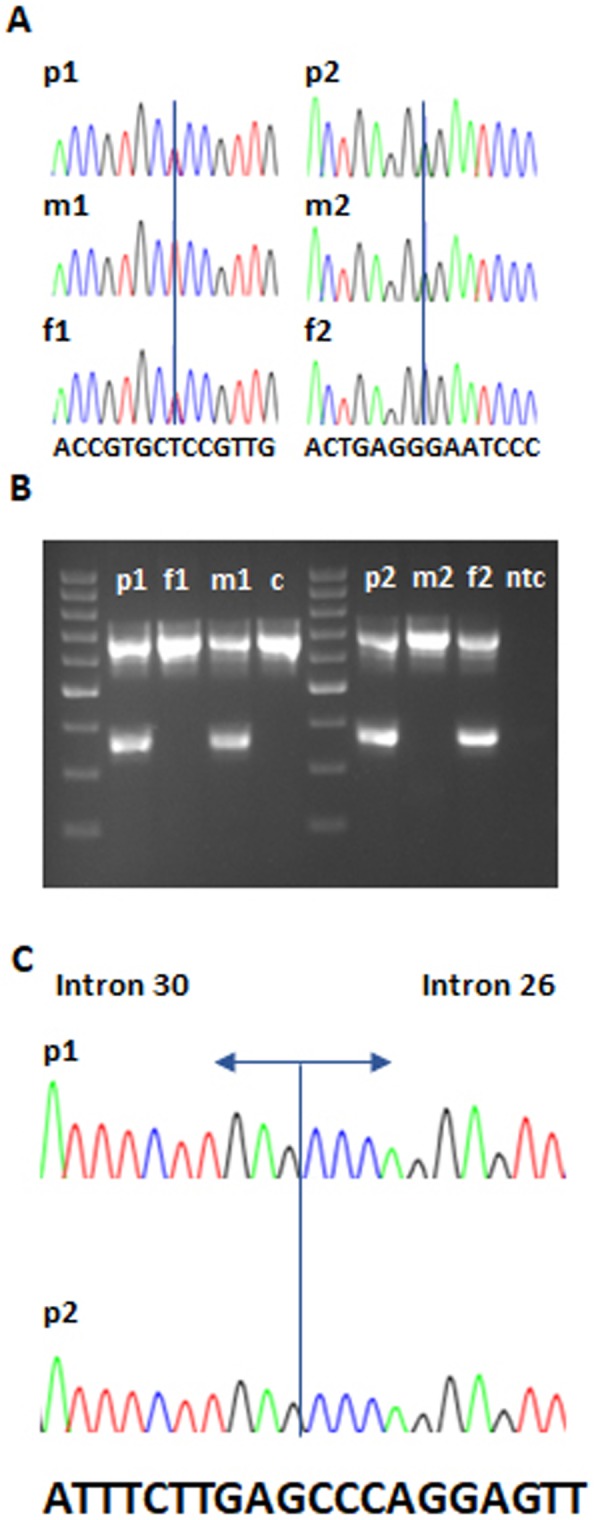
*IFT140* variants segregated with disease in both families with Sensenbrenner syndrome. Patient 1 has a heterozygous c.326 T > C (p.(Leu109Pro)) variant, which was inherited from the mother (**a**) and patient 2 has a heterozygous c.1565G > A (p.(Gly522Glu)) change, which was inherited from the father (**b**). Duplex-PCR revealed the presence of a tandem duplication p.Tyr1152_Thr1394dup in patients 1 and 2. This variant was inherited from the father in patient 1 and from the mother in patient 2, respectively (**c**). Sanger sequencing traces showing the breakpoints of the tandem duplication p.Tyr1152_Thr1394dup in both CED patients (**d**)

The p.(Gly522Glu) variant was listed in the Exome Aggregation Consortium database (ExAC) and has been reported in as a heterozygous variant in 17 out of 121,256 analyzed alleles, while this variant has been reported as a heterozygous variant in 39 out of 277,186 alleles in the gnomAD database. According to the ExAC and gnomAD databases (25.07.2019), the variant frequency is 0.01402% and 0.01407%, respectively. This change was not listed in the Exome Variant Server (EVS) database. In silico tools SIFT, PolyPhen, MutationTaster predict that this change has a deleterious effect on IFT140 protein. The variants was classified as likely pathogenic by ACMG guidelines [16]. In both families, segregation analysis of the identified variants was consistent with an autosomal recessive inheritance pattern of the disease (Fig. 3a, b), which provides further evidence for causality.

### Breakpoint analysis

Sanger sequencing of the breakpoints confirmed the presence of a tandem duplication spanning exons 27 to 30 of the *IFT140* gene in both patients. Breakpoint sequencing results are shown in Fig. 3c.

## Discussion

We identified compound heterozygous causal variants in *IFT140* in two unrelated Polish CED patients with early onset renal disease.

Mutations in the *IFT140* gene are associated with isolated retinal dystrophy, Mainzer-Saldino (MSS) Syndrome, Jeune Syndrome (JATD) and Opitz trigonocephaly C syndrome (OTCS) [13, 14]. JATD, MSS and CED are clinically overlapping disorders. *WDR35* and *IFT122* genes are most commonly mutated in Sensenbrenner syndrome, and variants in the *IFT140* gene are a rare cause of CED. Genetic analyses conducted in the present study revealed compound heterozygous variants in the *IFT140* gene in both unrelated CED patients. Both patients have a heterozygous tandem duplication p.Tyr1152_Thr1394dup on one allele in combination with a heterozygous missense variant on the second allele, p.(Leu109Pro), in patient 1, and a p.(Gly522Glu) variant in patient 2. Segregation analysis was performed in both families and the results were consistent with an autosomal recessive inheritance mode. Our results provide further support that the clinical spectrum associated with *IFT140* variants includes CED, which is important as current knowledge on the association of *IFT140* with CED is only marginal [8, 12].

The p.Tyr1152_Thr1394dup tandem duplication in *IFT40* is a known disease-associated variant that has recently been reported in eight families [12]. Seven patients of six of these families were diagnosed with Mainzer-Saldino syndrome, one patient had a phenotype reminiscent of Jeune syndrome and one patient had features of Sensenbrenner syndrome. This duplication is predicted to be in-frame and is thought to result in the addition of 243 amino acids within the tetratricopeptide repeat (TPR). RNA analysis showed that the duplicated exons are transcribed. However, western blotting did not detect the altered protein based on electrophoretic migration [12].

The missense variant p.(Leu109Pro) detected in patient 1 is a novel variant located in the WD40 functional domain of the IFT140 protein*.* In silico analyses based on SIFT, PolyPhen-2 and MutationTaster 2 showed that this change is predicted to disrupt the IFT140 protein and is likely pathogenic.

**Fig. 4:**
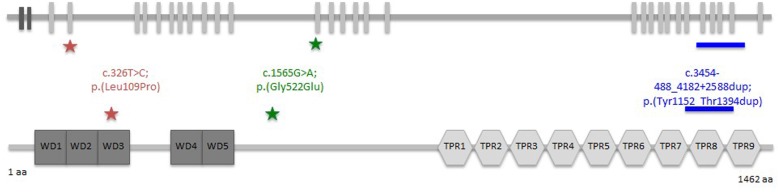
Localization of identified *IFT140* variants (Refseq NM_014714.4). Schematic representation of the gene structure of *IFT140*. Light grey blocks represent the exons (upper panel). The lower panel shows a schematic of IFT140 protein. The protein contains five WD repeats and nine tetratricopeptide (TRP) repeats. Stars and lines indicate the positions of the detected *IFT140* variants

The p.(Gly522Glu) change has been previously reported in a patient with Mainzer-Saldino syndrome and is predicted to have a deleterious effect on the IFT140 protein [9]. The location of the variants identified in both CED families is shown in a diagram of the *IFT140* gene and encoded protein in Fig. 4.

Both patients described here displayed early onset of renal disease. Patient 1 had a kidney transplantation at the age of 6 years and patient 2 started peritoneal dialysis at the age of 8 months with a recommendation for renal replacement due to an advanced stage of renal insufficiency. To date, only two CED patients harboring *IFT140* mutations have been described in literature: a male patient, reported by Bayat et al., who received a kidney transplant at the age of 4 years [8] and a female patient, described by Geoffroy et al., who developed end-stage renal failure, requiring dialysis at the age of 3.8 years and a renal transplant thereafter [12]. We conclude that progressive and end-stage renal disease may have an early onset in CED patients with *IFT140* variants.

Currently, there is no therapy available to significantly delay or prevent end-stage renal disease for patients diagnosed with autosomal recessive renal ciliopathies. Treatment options are limited to dialysis and transplantation. Typically, there is a 5–10 year interval between diagnosis of renal insufficiency and renal replacement, which provides a window of opportunity for therapeutic treatment of these patients. It is, however, challenging to develop therapies as generation of animal models is both laborious and costly given the enormous genetic heterogeneity that characterizes the renal ciliopathies. The low frequency of this group of disorders in the population is another complicating factor that impacts cost-effectiveness of therapy development based on animal models.

An alternate and more cost-effective approach is to assess potential therapeutic effects of drugs in the patient’s own cells. Induced pluripotent stem cells (iPSCs) technology may be a powerful tool to facilitate personalized drug assessment as iPSCs can be derived from fluids and tissues that are easy to obtain (blood, fibroblasts and urine) and can subsequently be differentiated into cells of a relevant tissue. Thus, a personalized approach for drug screening and gene-based therapy may be the future direction for individuals diagnosed with Sensenbrenner syndrome [17, 18].

The use of iPSCs is not only promising for personalized therapeutic assessments, it can also be helpful in making a diagnosis. For example, Forbes et al. recently described a patient with Mainzer-Saldino syndrome with compound heterozygous variants in *IFT140* for whom the iPSC technology was used to facilitate diagnosis. In this study renal organoids representing the developing nephron were created from patient-derived iPS cells (derived from skin-fibroblasts) in order to validate the clinical suspicion of a renal ciliopathy. These patient-derived organoids showed shortened and club-shaped primary cilia. This result is consistent with IFT140 dysfunction as loss of IFT140 function has been associated with shortened cilia and an accumulation of proteins at the ciliary tip [19]. By using proband-derived cell lines followed by rescue with CRISPR editing genetic differences between test and control lines can be reduced; Forbes et al. showed that CRISPR-Cas9 gene correction of *IFT140* could rescue the ciliary phenotype seen in their patient-derived organoids, thereby providing additional evidence that abnormal IFT140 caused the ciliary defects in this patient [11].

It has been suggested that gender plays a role in the severity of kidney dysfunction and that in males renal disease progresses more rapidly than in females, which implies that that sex hormones may be essential for direct or indirect modulation of the progression rate of renal disease [20]. It has been shown that kidney dysfunction is more often present in male CED patients with *IFT122* and *WDR35* than in female patients [3, 21, 22]. So far only one male and one female patient with *IFT140* variants were described in the literature and both developed early onset of renal disease and received a kidney transplant [8, 12]. Further clinical data is needed in order to confirm this possible gender effect in individuals with Sensenbrenner syndrome.

Signs of retinal disease have been reported in patients with variants in *IFT140* [12, 13]. Ophthalmological assessments of both patients presented in this study revealed nystagmus and hyperopia. In addition, patient 2 presented with strabismus. Similar ocular findings have been reported in a patient with features of MSS/CED with *IFT140* variants described by Geoffroy et al., while the patient described by Bayat et al. was diagnosed with retinal dystrophy showing that ocular defects are probably a frequent feature in CED patients with *IFT140* mutations [8, 12].

## Conclusions

In our study, we identified compound heterozygous *IFT140* variants in two CED patients using NGS combined with qPCR, duplex PCR and Sanger sequencing analysis. Without the use of qPCR and duplex PCR, one of the mutations p.Tyr1152_Thr1394dup would have been missed in each of our patients and they would have lacked a molecular diagnosis. Targeted analysis with these or comparable methods in unexplained CED patients who have a single heterozygous pathogenic variant in *IFT140* should therefore be considered. We found that individuals affected by Sensenbrenner syndrome and *IFT140* defects developed early onset end-stage renal disease. This finding may ultimately contribute to a better understanding of genotype-phenotype correlations in CED. This is important as not all CED patients develop rapidly progressive renal disease in early childhood and the molecular background of the variable kidney dysfunction seen in CED remains poorly understood. Finally, inter- and intrafamilial clinical variability further complicate the provision of an accurate prognosis for patients and their families [23]. Early monitoring of renal function is therefore warranted in patients with cranioectodermal dysplasia.

## Methods

### Molecular analysis

EDTA blood samples from the affected individuals and their parents were obtained for genetic analyses. DNA was extracted from peripheral leukocytes using a standard protocol.

### Whole-exome sequencing

Whole-exome sequencing was conducted on genomic DNA from patient 1. Exome enrichment was performed using a SureSelect Human All Exon kit v5 50Mb kit (Agilent, Santa Clara, USA) followed by sequencing on a HiSeq4000 system (Illumina, San Diego, CA, USA). Read mapping was performed using the Burrows-Wheeler Alignment Tool (BWA) and variants were called using the Genome Analysis Toolkit (GATK) haplotypecaller. Variant annotation was performed using the in-house annotation pipeline [24]. To prioritize variants in the sequencing data, variants that were non-genic, intronic (except for canonical spice sites) or synonymous were excluded. Variants that were present in the dbSNPv135 at a frequency of > 1% or > 1% in the in-house variants database were also excluded. A quality filter excluded variants with < 5 reads or < 20% variant reads.

### Targeted next-generation sequencing

A custom NGS gene panel encompassing 61 genes and 11 SNPs (225.709 kb) associated with craniofacial malformations was sequenced in patient 2. An additional file contains a list of genes and SNPs included in the NGS gene panel (see Additional file 1). NGS libraries were prepared using a SureSelect-based enrichment approach (Agilent Technologies) and these were subsequently sequenced on the Ion Torrent S5 platform. Reads were demultiplexed and aligned to the GRCh37 human reference genome using TorrentBrowser 5.0.4 software. The resulting BAM files were further processed using IonReporter 5.2. Estimates of coverage for individual genes were obtained by using bedtools 2.27.1 with a BED file defining coding parts of canonical transcripts. Variant quality control was based on four metric parameters; selection of variants with read depth ≥ 20, PHRED scores > 30 and variant frequencies of > 15%, and avoidance of strand-specific sequencing errors by excluding variants that occur with variant frequencies differing > 80% on opposite strands. In silico predictions based on SIFT, PolyPhen and PhyloP (46-way) were used to categorize the functional relevance of genetic variants and this information was retrieved from the IonReporter result files. In addition, CADD scores were evaluated, and bioinformatic prediction programs such as MutationTaster and SnpEff were used for variant classification. Population-specific allele frequencies were derived from Ensemble/VEP and gnomAD databases.

### Sanger sequencing

The presence of the through NGS identified missense variants was confirmed by Sanger sequencing. Primers were designed using Primer3. An additional file contains a list of primer sequences used for PCR and Sanger sequencing (see Additional file 2). PCR reactions in a total volume of 10 μl contained 1 μl of genomic DNA (100 ng/μl), 5 μl of 10x FailSafe Premix J buffer (Epicentre Biotechnologies), 0.5 μl of forward and reverse primer each (10 μmol/l), 2.9 μl of H_2_O and 0.1 μl of DNA polymerase (Taq DNA Polymerase, GenScript). PCR conditions were as follows: initial denaturation at 94 °C for 3 min followed by 35 cycles of denaturation at 94 °C for 15 s, annealing at 60 °C for 30 s, elongation at 72 °C for 45 s and a final elongation at 72 °C for 7 min. The PCR products were purified with Exonuclease I and shrimp alkaline phosphatase and sequenced using dye-terminator chemistry (kit v.3, ABI 3130XL) on an Applied Biosystems Prism 3700 DNA automated sequencer.

### Quantitative real-time PCR (qPCR)

A quantitative real-time PCR (qPCR) was performed using a ViiA™ 7 real-time thermal cycler (Applied Biosystems) in the index patients and their parents to detect abnormalities in dosage in *IFT140*. An additional file contains a list of primer sequences used for qPCR (see Additional file 2). qPCR reactions were run in triplicate in a total volume of 12 μl in each well and contained 6 μl of SYBR Green PCR Master Mix (Applied Biosystems), 5 μl of genomic DNA (2 ng/μl), and 0.5 μl of forward and reverse primer each (10 μmol/l). The following program was applied: stage 1: 95 °C for 10 min; stage 2: 95 °C for 30 s, 60 °C for 30 s, 72 °C for 1 min, for 40 cycles and stage 3: 95 °C for 15 s, 60 °C for 1 min and 95 °C for 15 s. The results were normalized to albumin gene (ALB) and the copy number of each of the targeted *IFT140* exons was determined by using a comparative DDCt method thereby using normal healthy control DNA as a calibrator. We performed sex determination of samples in reference to factor VIII gene (*F8*) located on X chromosome as a measure of quality assurance.

### Duplex PCR

In order to confirm the presence of *IFT140* tandem duplication, a duplex PCR was performed in both patients and their parents as described by Geoffroy et al. [12].

## Supplementary information

**Additional file 1.** List of genes and SNPs included in the NGS gene panel.

**Additional file 2.** List of primer sequences used for PCR, Sanger sequencing and qPCR.

## Data Availability

All data relevant to the study are included in the article or uploaded as supplementary information.

## Abbreviations

CED: Cranioectodermal dysplasia
ESRD: End-stage renal disease
ExAC: Exome Aggregation Consortium
IFT: intraflagellar transport
NGS: next generation sequencing
TRP: tetratricopeptide repeats
WES: whole exome sequencing
